# The effect of a unilateral upper extremity load (backpack) on the resulting spinal posture

**DOI:** 10.1186/1748-7161-8-S2-O26

**Published:** 2013-09-18

**Authors:** Patrick Knott, Sarah Davis, Ashley Harrison, Carly Larson

**Affiliations:** 1 Rosalind Franklin University of Medicine and Science, Chicago, USA

## Background

A frequent question from parents of adolescents is whether there are risk factors that could make a scoliosis curve increase, specifically whether carrying a heavy backpack on one shoulder could be detrimental to an existed curve. There has been very little research published to answer whether a unilateral upper extremity load would have an effect on spine position, let alone scoliosis progression (Chow, 2006).

## Purpose

The purpose of this study was to measure changes in spinal curvature with increasing unilateral carrying load in order to analyze the possible risk for patients with adolescent scoliosis.

## Method

Six young, non-scoliotic adults were measured with carrying loads of 15% and 20% of the subjects’ body weight compared to the neutral condition and analyzed for significant change. The formetric 3-dimensional/4-dimensional topography scanner was used to measure (1) weight distribution (2) kyphotic angle of the thoracic spine (3) lordotic angle of the lumbar spine (4) scoliosis angle of either the thoracic or lumbar spine, (5) coronal vertical axis (6) sagittal axis, and (7) shoulder tilt.

## Results

Coronal imbalance had a small shift of about 4 mm off center towards the side holding the backpack. Sagittal imbalance shifted forward by about 15mm, regardless of the side holding the backpack. The pelvic obliquity changed by about 2° away from the side holding the backpack. Kyphosis and lordosis stayed fairly stable. Shoulder slope changed by about 8°, with the backpack side lower, resulting in a 2mm shoulder height difference. Weight shifted towards the side holding the backpack by about 20%, but did not shift towards the front or back.

## Conclusions and discussion

We found that coronal imbalance, sagittal imbalance, shoulder tilt and weight distribution changed significantly from the neutral position with 15% and 20% weight applied to either side of the body. Although it is not known what effect this would have on progression of scoliosis, these changes could potentially contribute in a negative way to spinal imbalance.
